# Tanshinone IIA Attenuates the Inflammatory Response and Apoptosis after Traumatic Injury of the Spinal Cord in Adult Rats

**DOI:** 10.1371/journal.pone.0038381

**Published:** 2012-06-01

**Authors:** Xin Yin, Yue Yin, Fa-Le Cao, Yu-Fei Chen, Ye Peng, Wu-Gang Hou, Shu-Kai Sun, Zhuo-Jing Luo

**Affiliations:** 1 Institute of Orhopaedics Surgery, Xijing Hospital, Fourth Military Medical University, Xi'an, Shaanxi, P.R. China; 2 Department of Plastic Surgery, Xijing Hospital, Fourth Military Medical University, Xi'an, Shaanxi, P.R. China; 3 Department of Neurology and Respiration, The 222th Hospital of CPLA, Jilin, Jilin, P.R. China; 4 Department of Anesthesia, Xijing Hospital, Xi'an, Shaanxi, P.R. China; 5 Department of Operative Dentistry and Endodontics, School of Stomatology, Fourth Military Medical University, Xi'an, Shaanxi, P.R. China; National Institutes of Health, United States of America

## Abstract

**Background:**

Spinal cord injury (SCI), including immediate mechanical injury and secondary injury, is associated with the inflammatory response, apoptosis and oxidative stress in response to traumatic injury. Tanshinone IIA (TIIA) is one of the major extracts obtained from Salvia miltiorrhiza BUNGE, which has anti-inflammatory and anti-apoptotic effects on many diseases. However, little is known about the effects of TIIA treatment on SCI. Therefore, the aim of the present study is to evaluate the pharmacological action of TIIA on secondary damage and the underlying mechanisms of experimental SCI in rats.

**Methodology/Principal Findings:**

SCI was generated using a weight drop device on the dorsal spinal cord via a two-level T9-T11 laminectomy. SCI in rats resulted in severe trauma, characterized by locomotor disturbance, edema, neutrophil infiltration, the production of astrocytes and inflammatory mediators, apoptosis and oxidative stress. TIIA treatment (20 mg/kg, i.p.) after SCI induced significant effects: (1) improved motor function (Basso, Beattie and Bresnahan scores), (2) reduced the degree of tissue injury (histological score), neutrophil infiltration (myeloperoxidase activity) and the expression of astrocytes, (3) inhibited the activation of SCI-related pathways, such as NF-κB and MAPK signaling pathways, (4) decreased the production of pro-inflammatory cytokines (TNF-α, IL-1β, and IL-6) and iNOS, (5) reduced apoptosis (TUNEL staining, and Bcl-2 and caspase-3 expression) and (6) reversed the redox state imbalance.

**Conclusions/Significance:**

The results clearly show that TIIA has a prominent protective effect against SCI through inhibiting the inflammatory response and apoptosis in the spinal cord tissue after SCI.

## Introduction

Spinal cord injury (SCI) is a prevalent traumatic injury in the central nervous system (CNS) that leads to motor and sensory dysfunction in all ages. Trauma to the spinal cord causes immediate mechanical damage and a cascade of secondary damage [1]. After traumatic insult to the spinal cord, the tissues exhibit a progressive morphologic change, such as ischemia, hemorrhage, and edema [1], [2]. The secondary damage to the spinal cord develops within from minutes to hours following initial mechanical damage, and leads to a cascade of extracellular and intercellular events, including the infiltration of inflammatory cells, the destruction of neuronal and glial cells, neuronal dysfunction and death, and oxidative stress. Although great efforts have made to improve the outcome of patients with SCI, advances in therapy for this disease have been limited.

The pathophysiological mechanisms of SCI are complicated. During this period, inflammatory reactions are a crucial process of the secondary injury [3], [4]. Proinflammatory cytokines, such as TNF-α, IL-1β and IL-6, are primarily produced by inflammatory cells, and exist widely at the site of the lesion [1], [5]. Studies have demonstrated that the activation of MAPK signaling pathways following SCI is an important step in the inflammatory response [6]. Moreover, NF-κB is a major transcription factor that modulates the production of pro-inflammatory cytokines in the CNS [7], [8]. Previous studies have shown that apoptosis, which is regulated primarily through the caspase and Bcl-2 families [9], [10], is an important event in the secondary injury after SCI [11]. Reports indicate that apoptosis is involved in secondary degenerative damage, and eventually results in the functional disability of the spinal cord below the injured area. In addition, oxidative stress, which results in the imbalance of the redox state and contributes to the development of SCI. Because of the complex pathogenesis of SCI, a therapy that has multiple targets will be effective.

Tanshinone IIA (TIIA) is an important lipophilic diterpene extracted from Salvia miltiorrhiza BUNGE and is used in Chinese traditional herbal medicine (Danshen) for the treatment of many diseases, such as cardiovascular [12], [13], cerebrovascular [14], [15] and postmenopausal syndromes [16]. Previous studies show that TIIA might exert a series of biochemical effects through its anti-inflammatory [12], [16]–[18], anti-oxidative [19] and anti-apoptotic properties [20], and has been used to prevent ischemic injury [21]. However, whether TIIA produces protective effects to alleviate traumatic injury in the spinal cord is not yet clear.

In the present study, we aim to evaluate effects of TIIA on traumatic SCI. We used a weight drop device (NYU impactor) to create a rat SCI model to investigate a series of changes that occur in the spinal cord and to analyze the protective effects of TIIA. In our research, we found that pro-inflammatory factors were up-regulated after SCI, and subsequently the apoptosis process was activated. Moreover, our results demonstrated that TIIA markedly inhibited the expression of pro-inflammatory factors and related pathways, ameliorated the activation of apoptosis, and reversed the imbalance of the redox state.

## Materials and Methods

### Animal preparation

Sprague-Dawley male adult rats, weighing 220–250 g each, were obtained from the Animal Center (Fourth Military Medical University, Xi'an, China). The rats were kept in a temperature-controlled house under a 12 h light/dark cycle and fed a standard laboratory diet and water *ad libitum*. The Animal Care and Use Committee of the Fourth Military Medical University approved all experiments in accordance with the Declaration of the National Institutes of Health Guide for Care and Use of Laboratory Animals (Publication No. 85-23, revised 1985).

### Spinal cord injury

Spinal cord injury was induced using a weight drop device as previously reported [22]. Before this surgical operation, all instruments were autoclaved for sterilization. The acclimatization of rats was achieved in the surgical room prior to the start of the experiments. All animals were anaesthetized through intraperitoneal injection of 4% sodium pentobarbital (40 mg/kg, i.p.). An incision was made along the middle of the back, exposing the paravertebral muscles. A laminectomy was performed at the T9–T11 level, exposing the cord without damaging the dura. The exposed dorsal surface of the cord was subjected to weight drop impact using a 10-g metal rod at a height of 25 mm. Subsequently, the lesioned muscle and skin were sutured in layers, followed by a 1.0 ml subcutaneous injection of saline solution to replace the blood volume lost during the surgery. After the surgical operation, sterile gauze was wrapped around the wound. Animals were injected with penicillin (intramuscularly, 100,000 unit/per animal/day) to prevent post-operative infection. To relieve the pain from surgery, we subcutaneously injected buprenorphine (0.03 mg/kg) post-surgery for 3 days. During recovery from anesthesia, the rats were placed in a room with moderate temperature and humidity and covered with a warm blanket. A standard diet and water were provided daily to the rats ad libitum. Manual bladder expression was performed twice a day until the rats were able to urinate by themselves. Throughout the process, symptoms of infection, pain, decubitus, dehydration and autophagia were monitored daily.

### Experimental design

Rats were randomly divided into four groups, namely the sham group, sham/TIIA group, SCI group and SCI/TIIA group. We injected a 0.9% sterile saline (0.09 g NaCl dissolve 100 ml distill water) solution as a vehicle control. In the sham group, the animals were only subjected to laminectomy. In the sham/TIIA group, the animals were subjected to laminectomy with TIIA treatment. In the SCI group, the animals were subjected to SCI using an impactor and treated with an intraperitoneal injection of TIIA. TIIA was administered 1 h before operation (50 mg/kg). From day 1 to 7 post-SCI, TIIA was administrated (20 mg/kg) once a day at the same time. The current dose and timing of TIIA administration were applied on the basis of our preliminary experiments. At the indicated time point, the rats were sacrificed using cervical dislocation in all groups. Subsequently, the spinal cord was immediately exposed from T1 to T12, and the damaged tissue (T9 to T10) at the site of injury was cut. The time of sacrifice was determined according to the different parameters measured: motor function (BBB) score = 10 days after SCI; TUNEL staining = 3 days after SCI; western blot analysis for caspase-3 and Bcl-2 = 1, 3 and 7 days after SCI; real-time PCR analysis of proinflammatory cytokines = 6 h after SCI; and other measurements = 24 h after SCI.

### Behavioral examination

A motor function recovery test (n = 6) was performed twice a day from Day 1 to Day 10 after the spinal cord was contused. The behavioral test was scored in accordance with the rules of Basso, Beattie and Bresnahan (BBB) [28], which comprise 21 criteria for the movement of lower limbs, from complete paralysis to complete mobility. These criteria are based on the accurate observation of the lower limbs, including movement, step, and coordinated motor action. Two additional investigators blinded to treatment and grade observed the mechanically contused animals.

### Hematoxylin-eosin staining

The animals were sacrificed at 24 h after injury (n = 4 for each group) in all groups. Briefly, the rats were intracardially perfused with saline followed by treatment with ice-cold 4% paraformaldehyde. Subsequently, the spinal cord, including the center site of injury tissue, was collected. After fixation, the tissue blocks were embedded in paraffin, and divided into 5-µm slices. Microscopic evaluation was performed (Department of Pathology, Xijing Hospital, Xi'an, China) to characterize the spinal cord injury, and the slices were scored for edema, neutrophil infiltration, and hemorrhage. A scale of 0–4 represents the severity of spinal cord injury: 0 for none or minor, 1 for modest or limited, 2 for intermediate, 3 for widespread or prominent, and 4 for widespread and most prominent [26]. These tasks were conducted in a blinded manner, so that investigators were not able to identify the treatment and outcome of the animal until the completion of the measurement.

### Myeloperoxidase activity assay

The myeloperoxidase (MPO) activity, an indicator of polymorphonuclear leukocyte accumulation, was determined in the spinal cord tissue as previously described [27]. The extracted injured tissues from rats of all groups (n = 4 for each group) were weighed at 24 h after SCI, and the myeloperoxidase activity was measured. Each tissue sample was homogenized in homogenate medium [0.5% (w/v) hexadecyltrimethyl-ammonium bromide dissolved in 10 mM potassium phosphate buffer (pH 7)] and centrifuged at 20,000×g for 30 min at 4°C. An aliquot of the supernatant was subsequently incubated with a solution of 1.6 mM tetramethylbenzidine and 0.1 mM peroxide (H_2_O_2_). The rate of change in absorbance was measured spectrophotometrically at 460 nm. The MPO activity was defined as the quantity of enzyme required to degrade 1 mmol of H_2_O_2_ per min at 37°C, expressed as units of MPO/g wet tissue.

### Immunohistochemistry

Animals were sacrificed at 24 h after injury (n = 4 for each group) in all groups. Subsequently, tissue blocks were cut into 5-µm slices as mentioned above. After dewaxing and hydration, the tissue slices were incubated with citrate buffer (10 mM citric acid, 0.05% Tween 20, pH 6.0) for 10 min at 121°C to facilitate GFAP antigen retrieval. The sections were incubated in 1% H_2_O_2_ for 30 min at room temperature to inhibit endogenous peroxidase activity. After washing in PBS, the sections were preincubated with goat serum albumin for 30 min at 37°C followed by an incubation with a primary antibody against GFAP (Millipore, Bedford, MA, USA) for 18 h at 4°C. Subsequently, the sections were washed with PBS and stained with biotin-labeled rabbit anti-mouse IgG for 30 min at 37°C. The slices were washed with PBS and incubated in horseradish peroxidase-labeled streptavidin working solution for 30 min at 37°C. Finally, the slides were washed with PBS before application of diaminobenzidine (DAB), covered with a coverslip and analyzed under a light microscope.

### TUNEL staining

The rats were anesthetized with an intraperitoneal injection of 4% sodium pentobarbital (40 mg/kg) in all groups (n = 4 for each group) on Day 3 after SCI. The paraffin-embedded tissue slices were dewaxed, washed with PBS, and digested with proteinase K in a wet box for 30 min at 37°C. After washing with PBS, the slides were dipped in TUNEL reaction mixture, and incubated for 1 h at 37°C. After washing, the sections were incubated with converter-AP for 30 min at 37°C, and washed with PBS. Subsequently, the sections were stained with NBT/BCIP substrate solution for 1 h, and the signals were observed under a microscope. The cells with purple nuclei were considered dead. The number of TUNEL positive cells was counted.

### Extraction of RNA and Quantitative Real-time PCR

The rats were anesthetized with an intraperitoneal injection of 4% sodium pentobarbital (40 mg/kg) in all groups (n = 4 for each group) at 6 h after SCI. The lesioned tissue was dissected and homogenized in a solution of TRIZOL reagent (Invitrogen, Carlsbad, CA, USA). The RNA extraction was performed according to the manufacturer's instructions. The total RNA concentration was determined spectrophotometrically. A total of 1 µg of RNA was incubated with a reverse transcription mixture at 42°C for 50 min, followed by PCR amplification with the specific primers (synthesized by Invitrogen). We used the following primers: for TNF-α, P1 5′-TGATCGGTCCCAACAAGGA -3′ and P2 5′- TGCTTGGTGGTTTGCTACGA -3′; for IL-1β, P1 5′- TGCTGATGTACCAGTTGGGG -3′ and P2 5′-CTCCATGAGCTTTGTACAAG -3′; for IL-6, P1 5′- GCCCTTCAGGAACAGCTATG -3′ and P2 5′- CAGAATTGCCATTGCACAAC -3′; for iNOS, P1 5′- GCAAGT CCA AGTCTTGCTTGG-3′ and P2 5′-GGT TGATGAACTCAATGGCAT G-3′; for GAPDH, P1 5′- CCCCCAATGTATCCGTTGTG -3′ and P2 5′-TAGCCCAGGATGCCCTTTAGT -3′. The PCR conditions were 95°C for 15 s followed by 30 cycles of amplification consisting of 95°C for 5 s and 60°C for 30 s. The reactions were performed in triplicate, and the results were averaged. Each value was normalized to GAPDH mRNA levels to control for variations in the amount of input cDNA. The relative expression level of the genes compared with the control group was calculated using the ΔΔCt method.

### Extraction of protein and western blot

The tissues (n = 4 for each group) were homogenized in lysis buffer (10 mM Tris-HCl, 250 mM sucrose, 5 mM MgCl_2_, 2 mM EGTA, 1 mM phenylmethylsulfonyl fluoride, 2 mM sodium orthovanadate, 1 mM NaF, 1 mM leupeptin, 2 mM pepstatin A, 1 mM dithiothreitol)) and centrifuged at 4°C for 15 min at 900 g. Subsequently, the supernatant (total protein) was collected and the protein levels were quantified using a Coomassie brilliant blue assay. Total protein (50 µg) was separated on 12% SDS-polyacrylamide gels and transferred to nitrocellulose filter membranes. The membranes were labeled with primary antibodies (Millipore, Bedford, MA, USA) for phosphorylated or total NF-κB (1∶1000) and MAPKs (p38, ERK and JNK) (1∶1000), iNOS (inducible Nitric Oxide Synthase) (1∶400), caspase-3 (1∶200), Bcl-2 (1∶200), and β-actin (1∶5000) overnight at 4°C. The membranes were incubated with the appropriate horseradish peroxidase-coupled secondary antibodies (Santa Cruz Biotechnology, Inc., Santa Cruz, CA, USA) diluted at 1∶5000 for 1 h at 37°C followed by enhanced chemiluminescence detection of the proteins using an ECL kit (BestBio Inc, Shanghai, China). The protein density was quantified using imaging software (BioSens Digital Imaging 5, Shanghai Bio-Tech Inc, Shanghai, China). For the western blot analysis, caspase-3 and Bcl-2 were measured at 1, 3 and 7 days after SCI, and the other proteins were measured at 24 h after SCI.

### Biochemical analysis

To determine the cytokine levels (n = 4 for each group), the lesion site was rapidly dissected and homogenized in PBS at 24 h after SCI. After centrifugation at 4°C for 15 min at 900 g, the supernatants were used to measure the concentrations of TNF-α, IL-1β and IL-6 using corresponding ELISA kits (R&D systems, Minneapolis, MN), and the levels of malondialdehyde (MDA) and superoxide dismutase (SOD) using corresponding kits (Jiancheng Bioengineering Institute, Nanjing, China). The data are presented as the mean ± SEM from three replicate experiments.

### Statistical analysis

The data are expressed as the mean ± SEM, and the statistical analysis was performed with two-way analysis of variance (ANOVA) using the SPSS 13.0 software package (SPSS Inc., Chicago, IL), in which surgery (SCI vs. sham) and treatment (TIIA vs. vehicle) were the two fixed factors. A post-hoc analysis was performed using Dunnett's test for multiple comparisons. A significant difference was accepted as significant if p<0.05.

## Results

### Effects of TIIA on functional recovery after SCI

**Figure 1:**
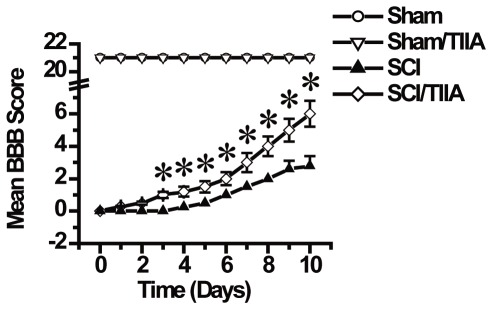
Effects of TIIA on functional recovery after SCI. The function of hindlimb recovery was accessed from day 1 to day 10 after SCI by Basso, Beattie and Bresnahan scores. The hindlimb dysfunction was ameliorated with treatment of TIIA (20 mg/kg). Bars represent means ± SEM. *p<0.05 vs. SCI group.

The hindlimb movements were abolished immediately after SCI. A spontaneous functional recovery after SCI in all groups was observed. As shown in Fig. 1, the rats in the SCI group gradually improved from Day 3, and rats in the SCI/TIIA group improved more rapidly. The statistical analysis showed a significantly greater increase in the BBB score from Day 3 to Day 10 in the SCI/TIIA group than in the SCI group. Ten days after SCI, the BBB average scores were 2.8±0.6 and 6±0.8 in the SCI and the SCI/TIIA groups, respectively (Fig. 1, p<0.05), indicating that TIIA treatment could promote the movement of rats with SCI.

### Effects of TIIA on histological changes after SCI

**Figure 2:**
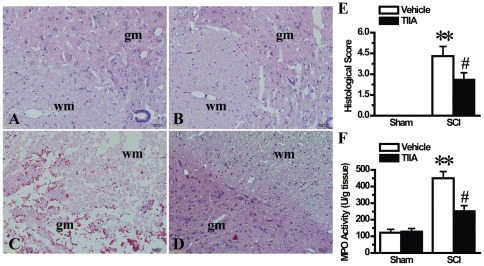
Effects of TIIA on histological changes at 24 h after SCI. Normal structure of spinal cord was observed in the sham (A) and sham/TIIA (B) groups at 24 h after SCI. A significant damage of spinal cord was shown by the presence of edema, haemorrhage and loose structure tissue at 24 h after SCI (C). A significant protective effect of TIIA (20 mg/kg) was observed in the SCI/TIIA group (D). wm: white matter; gm: gray matter. The histological score was accessed in a blinded manner (E). MPO activity in tissues was significantly increased at 24 h in the SCI group compared with the sham and sham/TIIA groups and was decreased with the treatment of TIIA (F). Bars represent means ± SEM. **p<0.001 vs. sham group, ^#^ *p*<0.05 vs. SCI group.

To evaluate the protective effect of TIIA on SCI, we observed histological changes in the spinal cord using electronic light microscopy at 24 h after operation. There were normal structures in both the white and grey matter in the sham and the sham/TIIA groups (Fig. 2A and 2B). The weight-drop rod was used to generate a mechanical insult, including edema, hemorrhage, neutrophil infiltration and loosened tissue structure in the SCI group (Fig. 2C). Compared with the SCI group, the morphologic changes were significantly alleviated in the TIIA-treated group (Fig. 2D), and some areas of the spinal cord even exhibited normal histological features. The calculated SCI scores are shown in Fig. 2E. TIIA exhibited a significant protective effect against SCI.

### Effects of TIIA on neutrophil infiltration after SCI

As previously described, the histological changes of SCI were associated with the influx of leukocytes into the spinal cord. Therefore, we studied the effect of TIIA on neutrophil infiltration using an MPO activity assay at 24 h after SCI. The results showed no significant difference in MPO activity between the sham and sham/TIIA groups (Fig. 2F), but the MPO activity was increased in the SCI group. On the contrary, the MPO activity was markedly decreased in the SCI/TIIA group (Fig. 2F).

### Effects of TIIA on the activation of astrocytes after SCI

**Figure 3:**
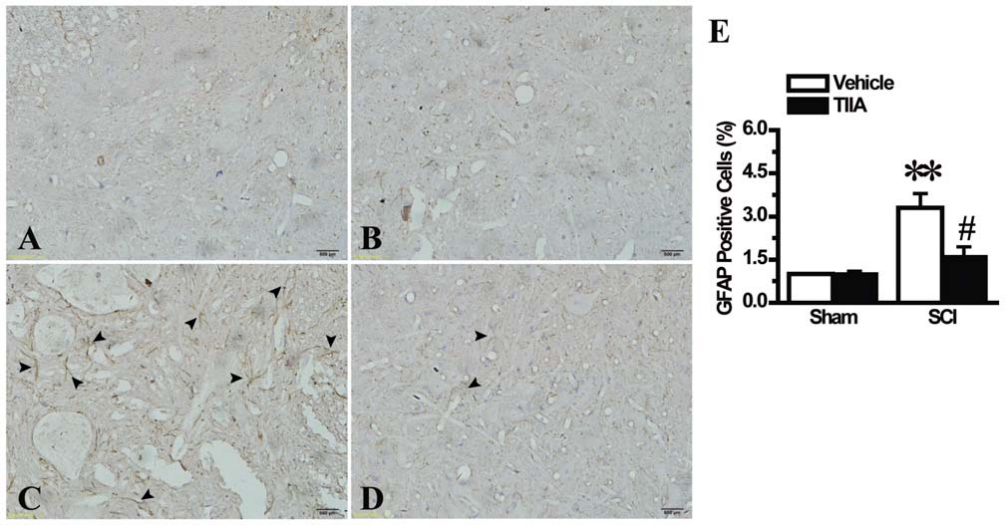
Effects of TIIA on the activation of astrocytes at 24 h after SCI. Few positive staining cells for GFAP were observed in the spinal cord tissues collected from the sham (A) and sham/TIIA (B) groups. A significant increase of GFAP positive cells was found in tissue from the SCI group at 24 h after injury (C). A significant reduction of GFAP positive cells was observed in the SCI/TIIA group (D). Analysis of the amount of GFAP showed the similar results (E). Bars represent means ± SEM. **p<0.001 vs. sham group, ^#^ *p*<0.05 vs. SCI group.

To test whether the activation of astrocytes in the injured tissues was changed, we measured GFAP positive cells under electronic light microscopy at 24 h after SCI. In both the sham and sham/TIIA groups, few GFAP positive cells were stained with GFAP antibody (Fig. 3A and 3B). The sections from the SCI group exhibited a significant increase in positive staining for astrocytes (Fig. 3C). However, as shown in Fig. 3D, TIIA treatment reduced the amount of GFAP-positive cells. The number of GFAP-positive cells was calculated in Fig. 3E.

### Effects of TIIA on NF-κB activation after SCI

**Figure 4:**
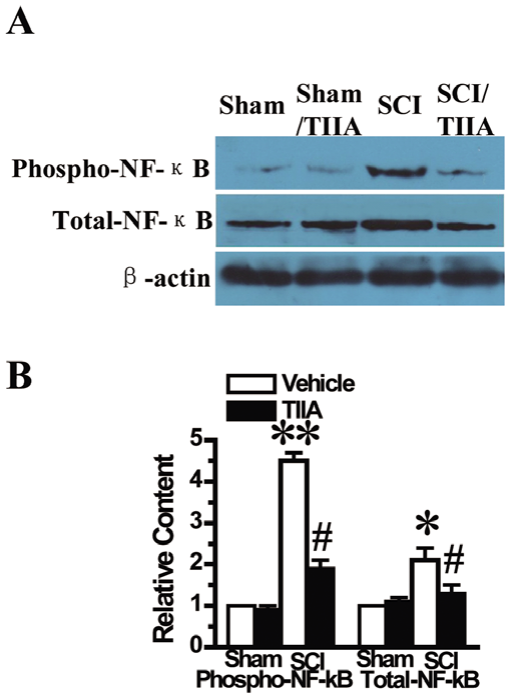
Effects of TIIA on NF-κB activation at 24 h after SCI. A basal expression of p-NF-κB and total NF-κB were detected by Western blot in the sham and sham/TIIA groups at 24 h after SCI, whereas NF-κB levels were increased after SCI. Treatment of TIIA (20 mg/kg) reduced the expression of NF-κB (A). The relative content of two types of NF-κB was counted (B). Bars represent means ± SEM. *p<0.05, **p<0.001 vs. sham group; ^#^ *p*<0.05 vs. SCI group.

As the NF-κB pathway plays a key role in inflammation-related injury, we investigated the level of phosphorylated NF-κB (p-NF-κB) and total NF-κB using western blot analysis to assess the therapeutic effect of TIIA at 24 h after SCI (Fig. 4A and 4B). In the sham and sham/TIIA groups, we detected a basal level of p-NF-κB and total NF-κB. The p-NF-κB was significantly increased and the total NF-κB was up-regulated in rats that were subjected to SCI. Moreover, we found that the expression of both forms of NF-κB was reduced in the SCI/TIIA group. As a result, we concluded that TIIA could inhibit the activation of the NF-κB pathway after SCI.

### Effects of TIIA on the activation of MAPK (p38, ERK, JNK) pathway after SCI

**Figure 5:**
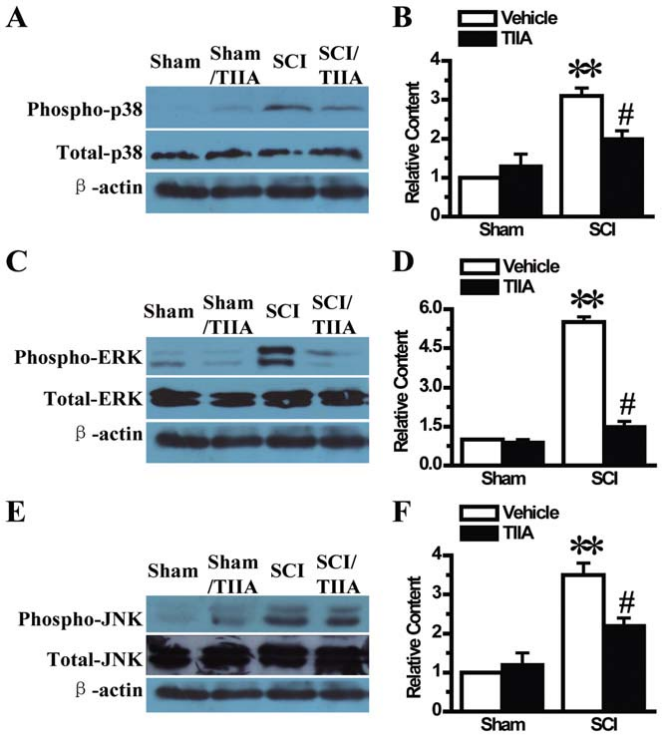
Effects of TIIA on MAPK pathway at 24 h after SCI. The expression of p-p38 was up-regulated in the SCI group compared with the sham and sham/TIIA groups. The level of p-p38 was attenuated with TIIA treatment as compared to the SCI group (A). The analyzed data for p-p38 are shown (B). The content of p-ERK was significantly increased in the SCI group at 24 h compared with the sham and sham/TIIA groups. TIIA prevented the expression of p-ERK (C). The analyzed data for p-ERK are shown (D). p-JNK was up-regulated after SCI, but a significant reduction of p-JNK in the tissues from the SCI/TIIA group was observed (E). The analyzed data for p-JNK are shown (F). Bars represent means ± SEM. **p<0.001 vs. sham group, ^#^ *p*<0.05 vs. SCI group.

Our study also measured the expression of p38, ERK and JNK in spinal cord tissue to fully investigate the mechanisms involved in the TIIA-mediated protective effects against SCI. The result showed that there were no differences in the activation of those pathways between the sham and sham/SCI groups at 24 h after operation (Fig. 5A and F). There was more p-p38 in the lesion site from the SCI group, but the up-regulation of p-p38 was significantly attenuated upon TIIA treatment (Fig. 5A and 5B). The changes in p-ERK and p-JNK expression were similar to that of p-p38 (Fig. 5C to 5F). Collectively, these data showed that TIIA could attenuate the activation of MAPK pathway after SCI.

### Effects of TIIA on the expression of pro-inflammatory cytokines after SCI

**Figure 6:**
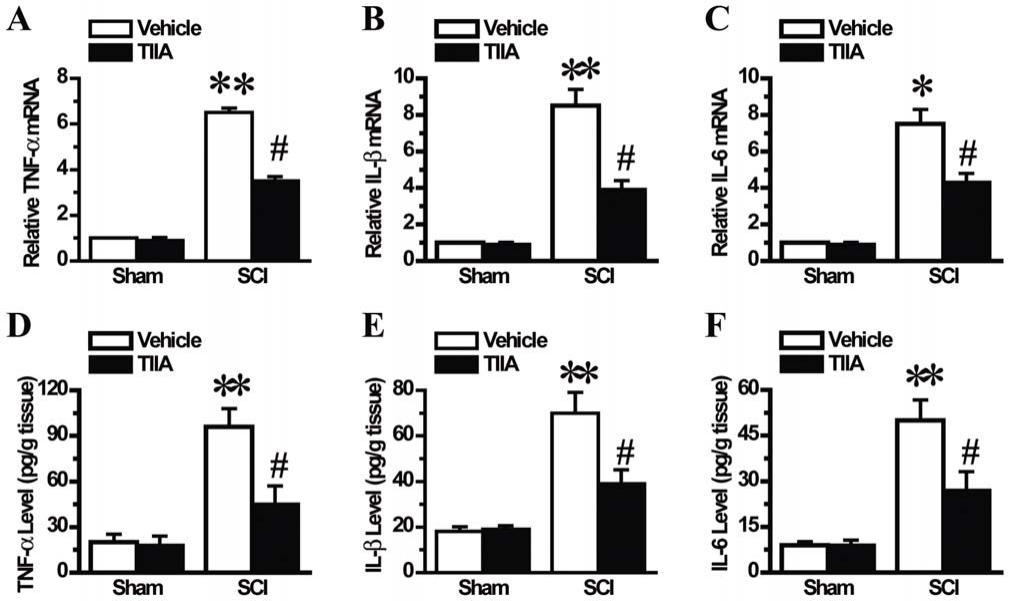
Effects of TIIA on the expression of pro-inflammatory cytokines at 6 h after SCI. The mRNA expression of TNF-α, IL-1β and IL-6 was enhanced in the tissues from the SCI group, but treatment of TIIA reduced their mRNA expression (A–C). Compared to the sham and sham/TIIA groups, the protein level of TNF-α, IL-1β and IL-6 was significantly up-regulated, whereas TIIA reduced the level of these proteins (D–F). Bars represent means ± SEM. *p<0.05, **p<0.001 vs. sham group; ^#^ *p*<0.05 vs. SCI group.

In our study, we evaluated the effect of TIIA on the expression of pro-inflammatory cytokines, such as TNF-α, IL-1β, and IL-6. In our preliminary experiment, we analyzed these cytokines at 6, 12 and 24 h after SCI. We found that the mRNA level of TNF-α, IL-1β, and IL-6 peaked at 6 h, and the concentration of these pro-inflammatory factors quickly decreased at 24 h after SCI (data not shown). Therefore, in this study, we measured the mRNA and protein expression of these cytokines from the damaged site at 6 h after SCI. As shown in Fig. 6, there were low levels of these cytokines in the sham and sham/TIIA groups. A significant increase in the mRNA and protein expression of TNF-α, IL-1β, and IL-6 was observed in the spinal cords of the SCI group (Fig. 6). Furthermore, compared with the SCI group, treatment with TIIA attenuated the expression of TNF-α, IL-1β, and IL-6 in the spinal cord.

### Effects of TIIA on the expression of iNOS after SCI

**Figure 7:**
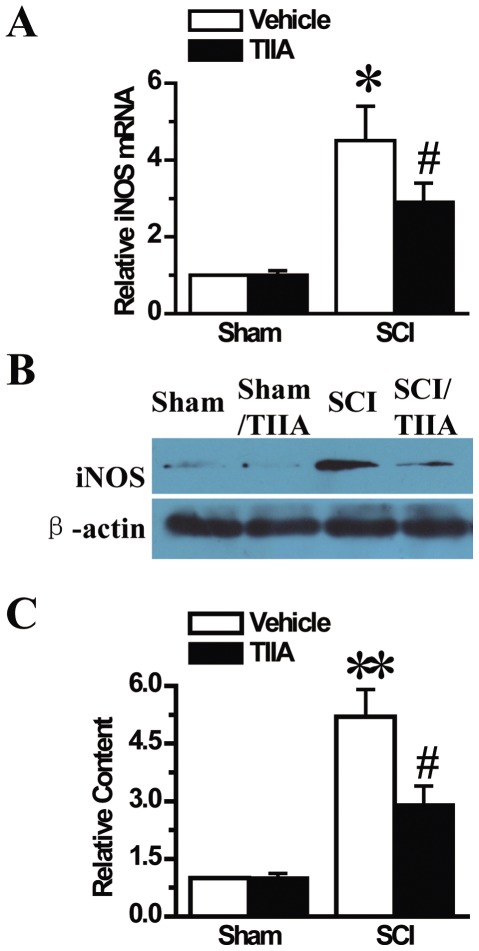
Effects of TIIA on the expression of iNOS at 24 h after SCI. RT-PCR results demonstrated that the expression of iNOS was increased after SCI. Treatment of TIIA decreased the mRNA level of iNOS compared to the SCI group (A). Western blot analysis demonstrated that the protein expression of iNOS was increased in the tissues from the SCI group, but the treatment of TIIA significantly reduced the level of iNOS (B). The analyzed data for relative iNOS protein content are shown (C). Bars represent means ± SEM. *p<0.05, **p<0.001 vs. sham group; ^#^ *p*<0.05 vs. SCI group.

iNOS is another inflammation-related mediator. We evaluated the expression of iNOS at 24 h after SCI. The results of the real-time PCR analysis showed that SCI caused a significant increase in iNOS mRNA expression in the SCI group (Fig. 7A). However, this increase was markedly downregulated upon the intraperitoneal injection of TIIA in the SCI/TIIA group (Fig. 7A). In addition, the results of the western blot analysis indicated that the SCI-induced iNOS protein expression was also decreased in the SCI/TIIA group (Fig. 7B and C).

### Effects of TIIA on apoptosis in spinal cord after SCI

**Figure 8:**
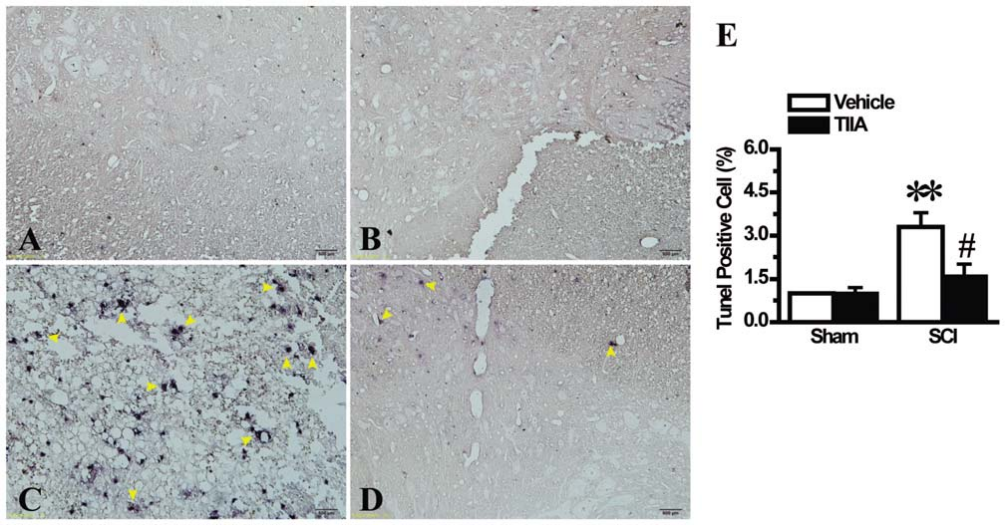
Effects of TIIA on apoptosis in spinal cord after SCI. Few apoptotic cells in the tissues from the sham and sham/TIIA groups were observed (A–B). SCI induced a marked increase of apoptotic cells at 3 days after trauma (C). In contrast, tissues obtained from TIIA treatement group showed significantly less apoptotic cells (D). The number of TUNEL positive cells was counted in 5 to 10 fields for each slide and the analyzed data are shown (E). Bars represent means ± SEM. **p<0.001 vs. sham group, ^#^ *p*<0.05 vs. SCI group.

To determine whether TIIA could affect SCI-induced apoptosis, we performed TUNEL staining on spinal cord sections. We found that the highest amount of apoptotic cells occurred at Day 3 after SCI in our preliminary experiment, thus we chose to analyze the cells in all groups at this time point. The results showed that a few TUNEL-positive stained cells were detected in the two sham groups (Fig. 8A and B). Moreover, SCI induced more TUNEL-positive cells in the grey and white matter in the SCI group (Fig. 8C), but TIIA resulted in a marked reduction in the number of these cells compared with the SCI group (Fig. 8D). The results of the quantitative analysis are shown in Fig. 8E. These data suggest that TIIA could reduce SCI-induced cell apoptosis in the spinal cord.

### Effects of TIIA on apoptosis-related factors after SCI

**Figure 9:**
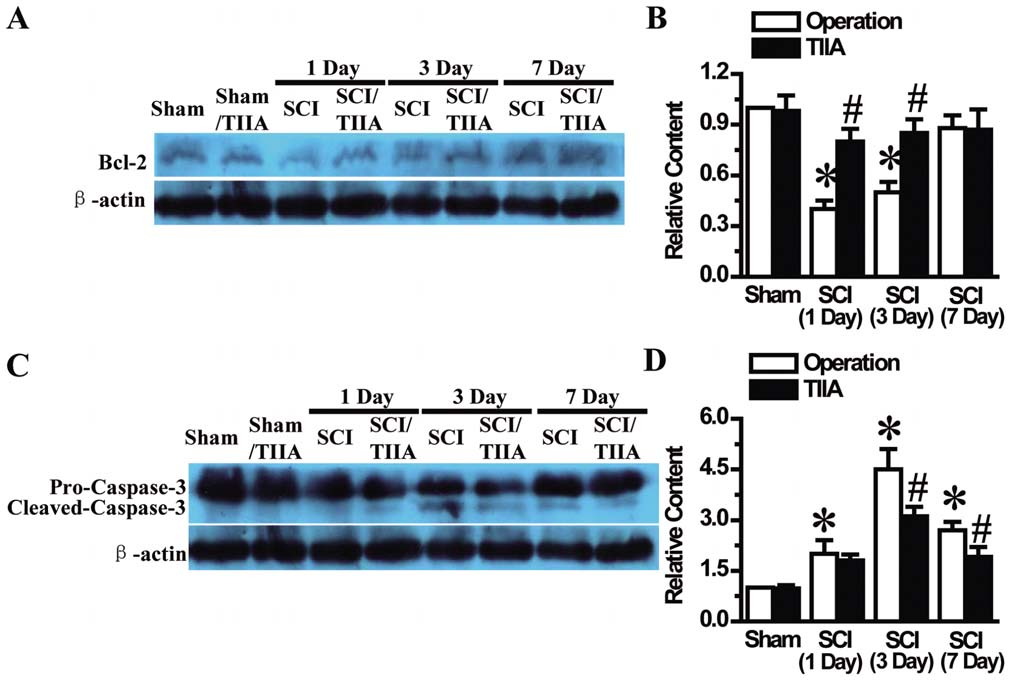
Effects of TIIA on apoptosis-related factors after SCI. The protein expression of Bcl-2 was reduced in the SCI group at 1 day and 3 days compared to the sham and sham/TIIA groups, whereas Bcl-2 was up-regulated in the SCI/TIIA group (A). The analyzed data are shown. (B) The cleaved caspase-3 expression began to increase at 1 day, peaked at 3 days and quickly reduced to a lower level at 7 days after SCI. However, TIIA treatment decreased the cleaved caspase-3 (C). The analyzed data are shown (D). Bars represent means ± SEM. *p<0.05 vs. sham group, ^#^ *p*<0.05 vs. SCI group.

To further confirm the effect of TIIA on apoptosis, we measured two apoptosis-related factors. First, Bcl-2, an inhibitive factor of apoptosis was subjected to western blot analysis. The results showed that SCI resulted in a significant reduction in the expression of Bcl-2 at Day 1 and Day 3 in the SCI group (Fig. 9A and B). However, in the TIIA/SCI group, the level of Bcl-2 was significantly increased compared with the SCI group (Fig. 9A and B). No difference in Bcl-2 expression was observed at the site of insult in the tissues on Day 7 after SCI (Fig. 9B). Second, we tested the change in caspase-3 expression, which is a component of the cysteine protease family that plays a key role in apoptosis. Compared with the rats in the sham and sham/TIIA groups, SCI induced a marked increase in caspase-3 at each time point (Fig. 9C and D). In contrast, cleaved caspase-3 was attenuated at Day 3 and Day 7 in the SCI/TIIA group compared with the SCI group (Fig. 9C and D).

### Effects of TIIA on oxidative stress markers after SCI

**Figure 10:**
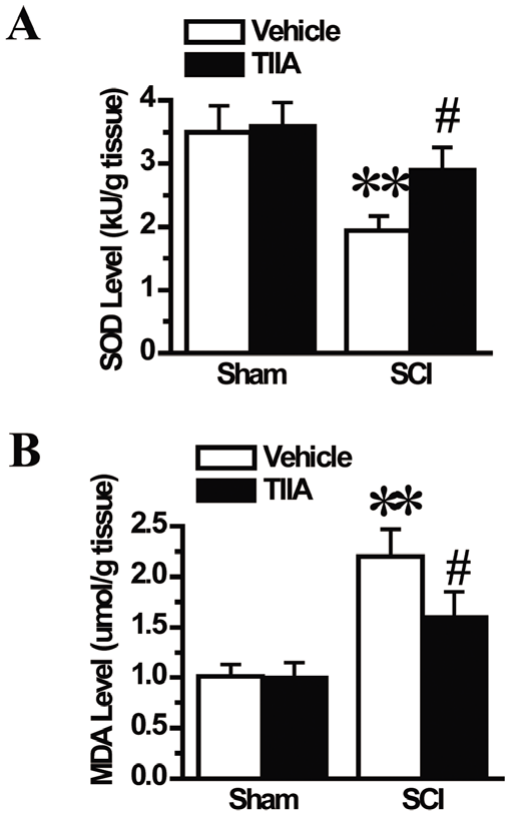
Effects of TIIA on oxidative stress markers after SCI. SCI induced the decrease of SOD activity at 24 h after SCI, but treatment with TIIA increased SOD 65 activity (A). The MDA level was significantly increased in the tissues from the SCI group at 24 h after SCI. TIIA treatment inhibited SCI-induced increase in MDA level (B). Bars represent means ± SEM. **p<0.001 vs. sham group, ^#^ *p*<0.05 vs. SCI group.

SOD and MDA are anti-oxidant and a pro-oxidant markers of oxidative stress, respectively, and were measured at 24 h after SCI. In the SCI group, SCI caused a significant decrease in the SOD activity and an increase in the MDA level. Compared with the SCI group, TIIA treatment after SCI dramatically rescued the SOD activity and significantly decreased the MDA level, indicating that TIIA played an important anti-oxidative role after SCI (Fig. 10A and B).

## Discussion

In this study, we demonstrated that TIIA exhibited a protective effect against SCI in rats. The administration of TIIA promoted motor function recovery of the hind limbs, ameliorated histopathological changes, inhibited the expression of proinflammatory factors and NF-κB and MAPK pathways, regulated SCI-induced apoptosis and apoptosis-related factors, and reduced the extent of oxidative stress in rats subjected to mechanical compressive insult by an NYU impactor.

To simulate the major events of SCI in rats, various experimental models have been developed [20]. One of the most widely used models is contusive injury. In this model, a rapid insult through dropping a weight onto the exposed surface of the dorsal spinal cord results in an immediate and transient contusion, which generates the primary damage. This device stimulates various levels of injury through correlative intensity weight, from moderate to severe, and even to complete paraplegia. The model's success is measured by the degree of movement in the bilateral hind limbs; a successful model occurs when the bilateral hind limbs have no movement [23]. In our experiment, we used this model to assess the therapeutic effect of TIIA on SCI.

Tanshinone (Danshen), is an herbal drug derived from the dried root of Salvia miltiorrhiza Bunge, and has been used either alone or in combination with other herbal materials to clinically prevent or treat many diseases in China and other countries [12], [21]. TIIA is one of the key components of Danshen. Previous studies suggest that TIIA might exhibit anti-inflammatory, anti-apoptotic and anti-oxidative capacities. We also assessed the therapeutic effects of TIIA in the SCI model. Based on the BBB score, TIIA promoted the recovery of locomotor function. Several studies demonstrate evidence of edema, hyperemia and incompact structures in the injured tissue [24]. We also observed the same results in the rat model of SCI, and found a significant effect of TIIA against SCI.

Astrocytes are activated upon SCI and are involved in the formation of a glial scar. Astrocytes also inhibit regeneration in the CNS. Moreover, activated astrocytes increase the production of intermediate filaments, such as GFAP (glial fibrillary acidic protein) and can secrete other molecules to restrict axonal growth. Together, this evidence showed that activated astrocytes exhibit a detrimental effect upon SCI [25]. Our data showed that GFAP-positive cells were increased after SCI, and TIIA administration significantly reduced the number of these positive cells. As a result, we conclude that TIIA effectively attenuated the number of activated astrocytes and glial scar formation.

Pro-inflammatory cytokines, such as TNF-α, IL-1β and IL-6, are key mediators and participate in the development of SCI [26], [27]. Nitric oxide (NO), which is mainly regulated through iNOS, also contributes to secondary damage after SCI [28]. Moreover, there are several pathways to modulate these inflammatory factors, such as NF-κB and MAPKs pathways [29]–[31]. Consistent with other studies [32]–[36], we detected increased mRNA and protein levels of pro-inflammatory cytokines and the activation of inflammation related pathways in SCI-induced damaged tissue. Moreover, we found that TIIA significantly reduced the levels of TNF-α, IL-1β, IL-6 and iNOS and markedly inhibited the activation of NF-κB and MAPKs pathways in the SCI model.

After injury, cell death occurs, and positive apoptotic cells are found at the lesion area, especially in the white matter [37]. Bcl-2 family proteins play an important role in mediating the intrinsic apoptotic pathway. Bcl-2 is an anti-apoptosis membrane protein, and prevents cell apoptotic processes through the inhibition of cytochrome c release from the mitochondria following the activation of caspase [38]. Caspases are a family of proteases that initiate apoptosis. Caspase-3 is a key factor in apoptosis. Various stimulators, such as Bcl-2 and Bax, regulate caspase activation [10], [39]. Caspase-3 participates in the downstream functions of the mitochondrial pathway, and the activation of caspase-3 might result in irreversible apoptosis. We found that cleaved caspase-3 was over-expressed in the SCI group, and TIIA could alleviate the effects of caspase-3. In addition, we found that treatment with TIIA increased Bcl-2 expression in our animal SCI model, suggesting that protection against apoptosis might be a prerequisite for regenerative processes in SCI.

Oxidative stress after SCI plays a pivotal role in secondary damage, and contributes to tissue injury during a post-traumatic inflammatory response and cell death [39]. During this process, the balance between anti-oxidants and pro-oxidants is disrupted. For example, SOD, which is a scavenger for reactive oxygen species (ROS), is consumed during oxidative stress. However, MDA, a lipid peroxidation end product, is increased during stress. Consistent with previous studies [40], we showed that SCI induced an increase in the expression of oxidative stress markers and reduced the expression of anti-oxidant markers. We also showed that TIIA treatment after SCI significantly reversed the changes of SOD activity and MDA levels, thereby restoring the balance of the redox state.

Taken together, we demonstrated that TIIA confers neuronal protection against apoptosis and attenuates the inflammatory and oxidative stress response after SCI. These data suggest that combination therapy against multiple targets might promote recovery from traumatic injury in the spinal cord. Although the mechanisms involved in TIIA protection against SCI in rats requires further elucidation, our data suggest that the application of TIIA might be an effective treatment for SCI and that TIIA could be widely used to alleviate SCI in humans in the future.
